# Impact of treatment delay on survival in women diagnosed with cervical cancer in Quito, Ecuador: a retrospective cohort study

**DOI:** 10.3332/ecancer.2025.2039

**Published:** 2025-11-19

**Authors:** Raul Puente-Vallejo, Alex García-Gutiérrez, Sebastián Jara-Jimenez, Martina Natalia Mosquera-Ruiz, Wilmer4 Tarupi

**Affiliations:** 1 Indoamérica Technological University, Quito 170301, Ecuador; 2 Metropolitan Hospital, Department of Radiotherapy, Quito 170521, Ecuador; 3 SOLCA Quito Centre, Teaching Department, Quito 170513, Ecuador; 4 SOLCA Quito Centre, Cancer Registry, Quito 170513, Ecuador

**Keywords:** uterine cervical neoplasms, time-to-treatment, survival, health planning

## Abstract

**Introduction:**

Delays in the treatment of cervical cancer may be associated with lower survival rates and a higher risk of disease progression. In low- and middle-income countries, the functioning of health systems may exacerbate this problem.

**Objective:**

To determine whether the initial point of care led to delays in the treatment of patients diagnosed with cervical cancer in Quito between 2012 and 2015, and to assess its impact on survival.

**Methods:**

A retrospective cohort study was conducted to analyse the survival of patients with cervical cancer treated at the SOLCA Quito Hospital. Patients were classified according to their initial point of contact (Group 1: initial care in the public health system with subsequent referral to SOLCA. Group 2: comprehensive care at SOLCA) and in relation to the International Federation of Gynecology and Obstetrics 2009 clinical staging at the time of diagnosis. The time to treatment initiation was compared using the Student’s t-test. Survival analysis was performed using Kaplan-Meier curves, estimating the probability of survival over time for both groups, stratified by clinical stage and performing an overall comparison of groups. In addition, the log-rank test was used to compare both survival curves and determine statistical significance using a significant value of p < 0.05.

**Results:**

A total of 1,363 cases were analysed. Group 1 had a mean waiting time of 162 days, while in group 2 it was 62 days to start treatment (p < 0.01). In terms of survival, group 2 versus 1 had a hazard ratio of 0.85 (95% confidence interval: 0.73–1). In terms of subgroups, the results favoured Group 2 in stages III and IV, the latter with an HR of 0.48 (95% confidence interval: 0.37–0.63) p < 0.01.

**Conclusions:**

Delays in the referral of cervical cancer patients from the public system led to delays in the start of treatment, affecting survival in patients with more advanced stages. Significant disparities in access to care highlight the need for future research aimed at identifying the causes of these delays and proposing possible interventions.

## Background

Cervical cancer is the second most common cancer and the second leading cause of cancer mortality among women in Ecuador. Data from 2022 indicate incidence rates (17.8 cases per 100,000 women) and mortality rates (8.9 deaths per 100,000 women) for the year, which are higher than the global average [1, 2]. In addition, the 5-year relative survival rate during the period 2010–2014 was 52% [2]. This figure contrasts sharply with high-income countries such as Japan, South Korea, Taiwan, Denmark, Norway and Switzerland, where survival rates reach levels close to 70%. In contrast, low- and middle-income countries (LMICs), such as many nations in Latin America, sub-Saharan Africa and Southeast Asia, report significantly lower survival rates and a higher burden of disease [3].

Cancer diagnosis and treatment in Ecuador take place within a fragmented healthcare system, divided into the public, social security and private sectors. Each sector is independent, with different financing, management and resource allocation mechanisms [3], where access to care depends on the individual’s ability to pay [4]. Although referrals between providers have been in place since 2013, when care is limited by factors such as capacity, timeliness or accessibility, this measure would generate additional administrative processes that contribute to delays and treatment of diseases. These delays, in turn, lead to more advanced stages of disease and higher mortality rates, as demonstrated by the 2013 evaluation of cancer care in Latin America [4, 5].

Several factors contribute to delays in the diagnosis and treatment of cervical cancer. These can be classified as patient-related, disease-related and external factors. Patient-related factors include advanced age (over 40 years), racial disparities (that affect Hispanic patients), low educational attainment and the presence of comorbidities [6–8]. Factors related to the disease include advanced stages of the disease, particularly metastatic disease, which would increase the likelihood of treatment delays. In addition, the toxicity associated with adjuvant therapies may also contribute to such delays [9]. External factors include late diagnoses due to delays in histopathological results or referrals, as well as long waiting times for medical care, lack of health insurance and high treatment costs [10–12].

These factors, although categorised operationally, can also be understood within the framework of the social determinants of health, which include structural dimensions such as socioeconomic inequality, segmentation of the healthcare system and geographical or cultural barriers that would condition access to timely cancer care [13–15].

Multiple studies have shown that timely therapy would improve 5-year survival rates compared to delayed treatment. Evidence shows that a delay of approximately 60 days after diagnosis reduces survival, and delays of more than four months double the risk of mortality [7, 16]. Likewise, it has been shown that delayed treatment would not only increase mortality but also increase the risk of disease progression; a delay of 4 months triples the risk of the tumour advancing to a more severe stage, negatively affecting overall survival [17, 18].

Although most studies highlight the negative impact of treatment delays on survival, others suggest that such delays would not have a prognostic impact, particularly in local treatments for early-stage cervical cancer [19, 20]. Taking the above into account, further research is needed to determine whether late initiation of treatment in conjunction with other factors, such as stage at diagnosis, reliably predicts survival outcomes in patients with cervical cancer.

The present study aims to examine treatment delays in patients diagnosed with cervical cancer in Quito between 2012 and 2015, according to the institution where the initial approach was performed, and to evaluate the impact of these delays on patient survival. Given that the SOLCA Quito Oncology Hospital would remain the main specialised cancer care centre in the city, where most patients are ultimately referred.

## Methods

A retrospective cohort study was conducted to analyse the survival outcomes of patients diagnosed with locally advanced cervical cancer in Quito. This included patients whose initial approach was in the public health system with subsequent referral to SOLCA (Group 1) for complementary treatment or those with an initial comprehensive approach at the SOLCA Oncology Hospital in Quito (Group 2). The primary outcome was defined as overall survival, understood as the time from diagnosis to death or the end of the follow-up period. Survival time was measured in months, considering patients alive at the end of the study period as censored. Therefore, survival time was calculated as the interval between the date of diagnosis and the date of death, loss to follow-up or until 31 December 2020, as the follow-up deadline.

### Cancer cases

Following approval by the ethics committee, anonymised data were obtained from the hospital registry of the SOLCA Quito Oncology Hospital, in compliance with ethical guidelines for retrospective studies. The following main variables were identified:

- Diagnosis: Cervical cancer.
- Treatment group: Patients whose first approach was in the Public Health System with subsequent referral for treatment at SOLCA (Group 1) versus patients with comprehensive management at the SOLCA Quito Oncology Hospital (Group 2).
- Clinical staging at the time of diagnosis: Classify according to the 2009 International Federation of Gynecology and Obstetrics (FIGO) system for cervical cancer (I–IV).
- Date of diagnosis
- Date of birth
- Vital status: Recorded as alive or deceased at the end of the follow-up period.
- Date of death

### Data analysis

The Student’s *t*-test was used to compare the time to treatment between the two groups. Survival analysis was performed using Kaplan–Meier survival curves to estimate the probability of survival over time in both treatment groups, stratified by clinical stage, and an overall comparison of the groups was also performed.

#### Log-rank test

The log-rank test was applied to compare the survival curves and determine the statistical significance of the differences between the two groups at each clinical stage. A *p*-value < 0.05 was considered statistically significant.

#### Estimation of hazard ratios (HR)

Although the Kaplan–Meier method does not provide direct estimates of HRs, HRs were inferred from the trends observed in the survival curves and the relative mortality risk of group 1 compared to group 2 was estimated by evaluating the divergence of the survival curves and their statistical significance. Multivariate analysis was performed using the COX model.

### Statistical software

The statistical analysis was performed using R Studio, a statistical software recognised for its robust capabilities for survival analysis. Survival packages were used to generate the curves and perform logarithmic tests. HRs were estimated from the curves and statistically significant *p*-values.

## Results

### Summary of findings

During the period analysed, 1,363 women diagnosed with cervical cancer were treated at the SOLCA Quito Hospital. Of these, 632 belonged to group 1 and 731 to group 2. The distribution of cancer stages is presented in Table 1.

### Time of treatment initiation

**Figure 1.**
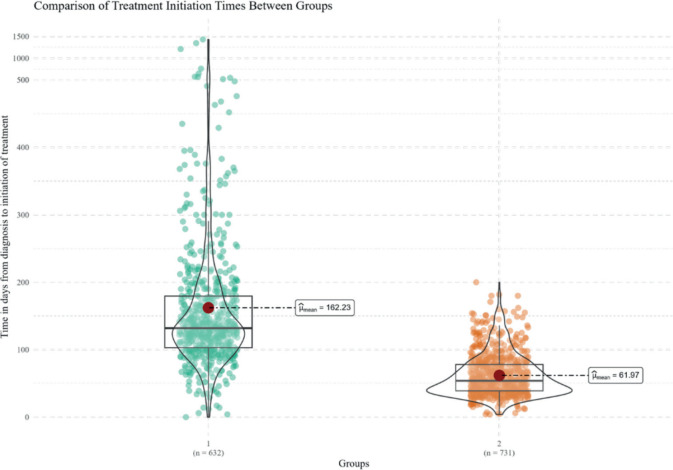
Comparison of time to treatment initiation between groups. Source: prepared by the authors based on study results.

The average time to treatment initiation, defined as the administration of any oncological therapeutic modality (surgical, radiotherapy or systemic), was 162 days for Group 1 and 62 days for Group 2. The test revealed a statistically significant difference between the two groups (*p* = 0.004) (Figure 1).

### Survival analysis

**Figure 2.**
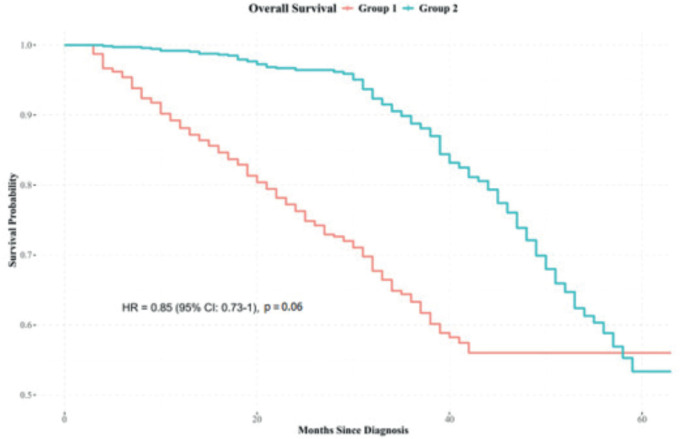
Overall survival analysis combining all stages. Source: prepared by the authors based on study results.

Kaplan-Meier survival analysis indicated differences in survival outcomes between Groups 2 and 1, with an HR: 0.85 (95% confidence interval: 0.73–1), *p* = 0.06 (Figure 2).

**Figure 3.**
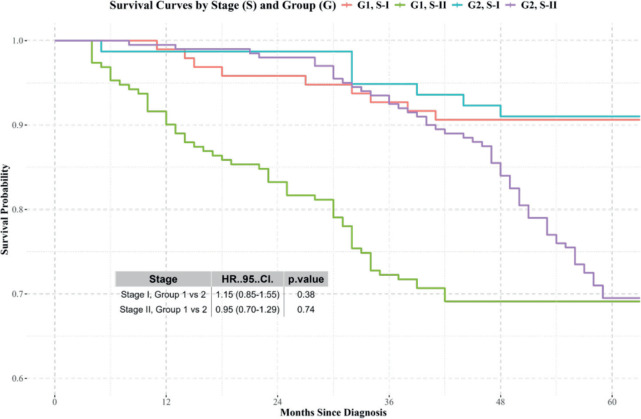
Kaplan-Meier survival curves for patients diagnosed and treated at SOLCA (Group 2) versus those diagnosed externally and treated at SOLCA (Group 1) for stage I and II cervical cancer. Source: prepared by the authors based on study results.

**Figure 4.**
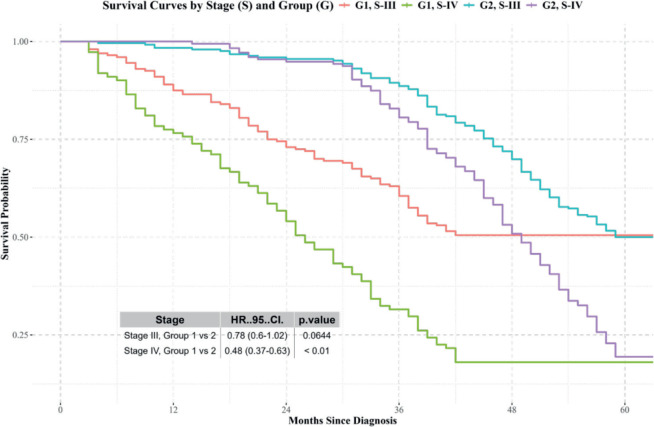
Kaplan-Meier survival curves for patients diagnosed and treated at SOLCA (Group 2) versus those diagnosed externally and treated at SOLCA (Group 1) for stage III and IV cervical cancer. Source: prepared by the authors based on the study results.

- Stages I and II: No significant differences in survival were observed between the groups (Figure 3).
- Stages III and IV: Differences in survival were observed, with Group 2 showing better results (Figure 4).

No significant differences were identified in age at diagnosis and survival between the two groups: HR: 0.99 (95% confidence interval: 0.99–1.04), *p* = 0.69. The results were further stratified by clinical stage at diagnosis.

## Discussion

The findings of this study highlight the significant impact of treatment delays on survival outcomes in patients with advanced cervical cancer, particularly those with FIGO stage IV disease. Patients initially treated in the public health system experienced substantially longer delays in treatment initiation compared to those who would have been initially treated and managed at the SOLCA Quito Oncology Hospital. These disparities in timely access to care reflect global evidence that identifies socioeconomic, geographic and systemic barriers as key determinants of cancer outcomes [7, 21].

Cervical cancer remains a major public health challenge, particularly in low- and LMICs, where structural inequalities limit access to early diagnosis and timely treatment. LMICs face significant barriers associated with fragmented health systems, poor oncology infrastructure and limited human resources. In this analysis, countries will be compared according to their income level, following the World Bank classification, which would allow for a comparison of Ecuador’s situation with that of other countries with similar incomes in different regions, including Asia, where significant gaps in cervical cancer care also persist. In this study, the delays observed among patients diagnosed within the public system are concerning, as starting treatment within 60 days of diagnosis would be crucial for improving survival [17].

A Brazilian study reported similar findings, observing that prolonged delays from diagnosis to treatment initiation, particularly beyond 120 days, were associated with worse outcomes in Brazilian women [7]. The delays in our cohort, which would average more than 100 days for patients in the public health system, not only reflect resource constraints but also highlight the systemic inefficiencies present in many LMICs, where the diagnostic and treatment infrastructure is insufficient to meet demand [21].

The impact of these delays on survival was further confirmed by our survival curves, which would demonstrate a strong association between treatment delays, place of diagnosis and survival in patients with advanced stages. The lower survival rates observed in patients diagnosed within the public system may be related to advanced stage at diagnosis, as delayed access to care often results in disease progression before treatment is initiated.

This finding is consistent with a study that would report that advanced-stage diagnosis and prolonged waiting times were significant predictors of reduced survival in patients with cervical cancer [22]. In their study, women with advanced-stage cervical cancer (FIGO III and IV) had a significantly lower survival rate than those diagnosed at earlier stages—a finding that underscores the importance of timely diagnosis and early intervention. This underscores not only the importance of timely diagnosis and early intervention but also their direct impact on improving patient survival.

In our study, delays in treatment among patients in the public system compared to those initially treated at the SOLCA Oncology Hospital likely contribute to the delays observed and worse outcomes for Group 1. This suggests that even in settings where treatment protocols are standardised, inequities in access to medical care would undermine survival rates. In this regard, it has been reported that women with cervical cancer who would start radiotherapy between 120 and 179 days after diagnosis were three times more likely to experience tumour progression to a more advanced stage. Those who would start treatment after 180 days faced an even greater risk compared to those who would start within 60 days of a confirmed diagnosis [16]. A study conducted in Ethiopia found that women who would start radiotherapy between 120 and 179 days after diagnosis had a relative risk (RR) of clinical progression of 3.1 times (95% confidence interval: 1.8–5.3), while those who would start after 180 days had an RR of 4.1 times compared to patients treated within the first 60 days (95% confidence interval: 2.3–7.1) [27].

Similarly, a cohort study in the United States showed that delays of more than 90 days from diagnosis to treatment initiation would be associated with a 12% reduction in 5-year overall survival compared with those who started treatment in a timely manner. In Brazil, a study reported that patients with delays of more than 120 days would be 2.6 times more likely to progress to advanced clinical stages and would have an adjusted HR of 1.9 for death compared with those who started treatment within the first 2 months. Furthermore, Ramey *et al* [17] demonstrated that a delay of more than 60 days would be associated with a 31% increase in the risk of death (HR: 1.31; 95% confidence interval: 1.10–1.56), even after adjusting for clinical stage, age and comorbidities.

Furthermore, the role of patient delay, defined as the interval between symptom onset and diagnosis, is a crucial consideration in understanding treatment delays [23]. In our cohort, many patients likely experienced significant delays in seeking care, particularly those in the public system, where access to screening and diagnostic services is limited [24].

A study conducted in 2023 identified similar patterns of delay in young women with cervical cancer in China, where delays were often exacerbated by factors such as lack of knowledge about the disease, limited access to healthcare facilities and financial constraints [23]. In our study, the longer delays observed in the public system may partially reflect these same barriers, suggesting a need for greater emphasis on early detection programmes and public health education to improve timely access to care.

According to National Institute of Statistics and Census of Ecuador data for 2015, the year of diagnosis for the cohort participants, the average level of schooling in the Metropolitan District of Quito was 10.8 years. This value is above the national average, which would be 10.2 years [28]. According to a UN Women report, Afro-Ecuadorian women have an average schooling of 10.2 years, indigenous women 8 years and Montubia women 7.3 years [29]. The central theme of all these studies, including ours, is the profound impact of structural inequalities on cancer outcomes. Patients in LMICs, such as Ecuador, face significant challenges in accessing timely, high-quality care. These challenges are exacerbated by the lack of comprehensive screening programmes, insufficient diagnostic infrastructure, shortages of specialised human resources and lengthy referral processes, all of which contribute to delays in treatment initiation [23, 25].

This study highlights the urgent need for reforms in the healthcare system to reduce delays in treatment and improve access to timely care. Strengthening public health infrastructure, particularly in terms of diagnostic capacity and referral networks, is essential. In addition, expanding public awareness campaigns to promote early detection and reduce patient delays is crucial to improving cervical cancer outcomes. Interventions targeting disparities in access to healthcare, particularly for marginalised populations, can help reduce gaps in treatment initiation and survival [14, 20, 22].

## Conclusion

This study provides evidence that delays in treatment initiation have a negative effect on the survival of patients with advanced cervical cancer. Significant disparities in access to cancer care were identified, highlighting the urgent need to implement targeted interventions that address the structural barriers in the healthcare system that would contribute to such delays. Future research would analyse the underlying causes of treatment delays and evaluate strategies aimed at improving timely and equitable access to specialised care.

### Limitations

The retrospective nature of the design, possible selection and information biases and the unavailability of certain clinical and socioeconomic variables that would influence survival.

## Conflicts of interest

The authors declare no conflicts of interest.

## Funding

This research did not receive external funding.

## Ethical approval

The study was conducted in accordance with the Declaration of Helsinki and was approved by the Human Research Ethics Committee of the SOLCA Quito Cancer Hospital. Reference number: CEISHSOLCAQ.OBS 19.125. Date of approval: November 2019.

## Data availability statement

This article is based on anonymised data on cancer cases provided by the hospital registry. The database used to support the findings of this study may be available upon request by contacting the corresponding author or writing to rnt@solcaquito.org.ec.

## Author contributions

PRV: Conceptualisation, methodology, validation, data curation, writing – review and editing, project management.

AGG: Methodology, software, formal analysis, writing – review and editing.

SJJ: Research, writing – original draft.

MNMR: Resources, writing – original draft.

TW: Conceptualisation, validation, writing – review and editing, supervision.

## Send language

Spanish.

## Figures and Tables

**Table 1.** Descriptive characteristics of the main variable by treatment group. Source: Prepared by the authors.

Variable	1		2		*p*-value^1^	
Age at diagnosis (years)	52.3		53.3		0.2	
Overall survival (mean in days)	162.2		61.9		0.004	
Stage	1		2		Total	0.02
	*N*	%	*N*	%		
I	134	55%	111	45	245	0.901
II	188	49	197	51%	385	0.37
III	200	45	248	55%	448	0.06
IV	110	39	175	61%	285	<0.001
Total	632	46	731	54%	1363	
Vital status	1		2		Total	0.4
	*N*	%	*N*	%		
Alive	354	56	390	53	744	
Deceased	278	44%	341	47%	619	
Total	632	100	731	100	1363	

a Welch’s *t*-test for two samples; Pearson’s chi-square test
